# Identifying variation for N-use efficiency and associated traits in amphidiploids derived from hybrids of bread wheat and the genera *Aegilops*, *Secale*, *Thinopyrum* and *Triticum*

**DOI:** 10.1371/journal.pone.0266924

**Published:** 2022-04-15

**Authors:** Ajit Nehe, Julie King, Ian P. King, Erik H. Murchie, M. John Foulkes

**Affiliations:** Division of Plant and Crop Science, School of Biosciences, University of Nottingham, Loughborough, Leicestershire, United Kingdom; Institute of Genetics and Developmental Biology Chinese Academy of Sciences, CHINA

## Abstract

Future genetic progress in wheat grain yield will depend on increasing biomass and this must be achieved without commensurate increases in nitrogen (N) fertilizer inputs to minimize environmental impacts. In recent decades there has been a loss of genetic diversity in wheat through plant breeding. However, new genetic diversity can be created by incorporating genes into bread wheat from wild wheat relatives. Our objectives were to investigate amphidiploids derived from hybrids of bread wheat (*Triticum aestivum* L.) and related species from the genera *Aegilops*, *Secale*, *Thinopyrum* and *Triticum* for expression of higher biomass, N-use efficiency (NUE) and leaf photosynthesis rate compared to their bread wheat parents under high and low N conditions. Eighteen amphidiploid lines and their bread wheat parents were examined in high N (HN) and low N (LN) treatments under glasshouse conditions in two years. Averaged across years, grain yield reduced by 38% under LN compared to HN conditions (P = 0.004). Three amphidiploid lines showed positive transgressive segregation compared to their bread wheat parent for biomass per plant under HN conditions. Positive transgressive segregation was also identified for flag-leaf photosynthesis both pre-anthesis and post-anthesis under HN and LN conditions. For N uptake per plant at maturity positive transgressive segregation was identified for one amphidiploid line under LN conditions. Our results indicated that introgressing traits from wild relatives into modern bread wheat germplasm offers scope to raise biomass and N-use effciency in both optimal and low N availability environments.

## 1. Introduction

Bread wheat has been selected in plant breeding for improved grain yield and adaptability to diverse environments and agriculture practices. This has led to the loss of genetic diversity [1]. and modification in traits like vernalization requirement and photoperiod requirement. Development of *Rht* semi-dwarf wheat cultivars that responded to higher N fertilizer doses in the 1960s and 1970s, the so called “Green Revolution varieties”, also narrowed the genetic base [2]. Moreover, wheat is a naturally self-pollinated crop with less heterozygosity and heterosis than outcrossing crops [3]. Therefore, hybrid seed production to enhance diversity and break the yield plateau is not straightforward.

However, new genetic diversity can be created by incorporating genes into bread wheat cultivars from wild relatives [4, 5], which have been shown to contain variation for traits of agronomic importance. For example, *Triticum urartu* (wheat A genome donor) has been implicated in photosynthetic capacity [6, 7], *Thinopyrum bessarabicum* is a highly salt tolerant species [8], and. *Aegilops speltoides* (the putative wheat B genome donor) is adapted to drought/heat environments and nutrient-poor areas [9]. Furthermore, the use of ancestral-derived germplasm, introgressing genes from the ancestral wheat species, has been suggested as a source of variation for tolerance of low N availability in wheat breeding programs [10, 11]. Wheat wild relatives can be crossed to bread wheat to produce an interspecific hybrid or amphihaploid. The amphihaploid is then chromosome doubled (e.g. using colchicine) to produce an amphidiploid containing both the complete wheat genome and the complete genome from the wild relative [4].

N-use efficiency (NUE) can be defined as the grain dry matter (DM) yield (kg DM ha^−1^) divided by the supply of available N from the soil and fertilizer (kg N ha^−1^; [12]) and can be divided into two components: (i) N-uptake efficiency (NUpE; above-ground N uptake per unit N available) and (ii) N-utilization efficiency (NUtE; grain dry matter yield per unit above-ground N uptake). Wild relatives of bread wheat have been reported to have higher leaf photosynthetic rates compared to modern cultivars [13–15] suggesting that wheat breeding may have resulted in lower photosynthetic rates. Austin et al. [6] reported that the rate of leaf net photosynthesis was in general highest in the diploid wheat species, intermediate for the tetraploid species and lowest for hexaploid *T*. *aestivum*. Synthetic lines (*Triticum durum* × *Aegilops tauschii*) and derivatives developed by CIMMYT have been associated with higher leaf photosynthetic rate [16] and grain yield [17] than the recurrent bread wheat parents under optimal conditions. In addition, primary synthetic spring wheats have been shown to have greater root biomass compared to recurrent parents in Australia [18] and CIMMYT synthetic-derived wheat lines expressed increased partitioning of root mass to deeper soil profiles and grain yield under drought compared with the recurrent bread wheat parents in NW Mexico [19, 20].

Increasing biomass of the wheat crop for future gains in yield potential implies an additional requirement for N capture to support photosynthesis. Increased N fertilizer inputs, however, will have economic implications as well as environmental impacts, through nitrate leaching into groundwater and conversion of nitrate by denitrifying soil bacteria into nitrous oxide, a greenhouse gas which contributes to global warming [21, 22]. The development of cultivars with reduced requirements for N fertilizer will therefore be of economic benefit to farmers and help to reduce environmental contamination associated with inputs of N fertilizers [23, 24]. Promising traits for selection by breeders to increase NUE include deeper roots for increased N uptake [25], increased leaf photosynthesis rate [11] along with the stay-green trait associated with optimized post-anthesis N remobilization [26] and/or late N uptake [27, 28].

In the present study, eighteen amphidiploid lines were characterized for NUE and associated traits including plant biomass and leaf photosynthesis rate in glasshouse experiments. The amphidiploid lines were produced by crossing accessions of the wild relatives of wheat (*Amblyopyrum muticum*, *Aegilops speltoides*, *Aegilops umbellalata*, *Aegilops comosa*, *Thinopyrum turcicum* and *Thinopyrum bessarbicum*) with the bread wheat cultivars, Chinese Spring, Paragon, Pavon 76 and Highbury [4]. The objectives were to: (i) identify novel wheat lines (amphidiploids) expressing higher biomass, NUE and leaf photosynthesis rate than the bread wheat parents under high and low N conditions and (ii) understand the physiological mechanisms underlying the improved performance in the novel ancestral wheat-derived amphidiploid lines compared to bread wheat parents.

## 2. Materials and methods

### 2.1 Plant materials

Eighteen amphidiploid lines along with four bread wheat parental cultivars were grown in each of two glasshouse experiments (Table 1). The bread wheat parent cultivars were Paragon (PAR), Highbury (HB) (UK spring wheat cultivars), Pavon 76 (PAV) (Mexican CIMMYT spring wheat cultivar) and Chinese Spring (CS) (Chinese spring wheat landrace). The amphidiploids were produced at the Wheat Research Centre, University of Nottingham by crossing bread wheat as the female parent with a wild species to produce a F_1_ interspecific hybrid or amphihaploid. The F_1_ hybrids were then chromosome doubled using colchicine to produce the amphidiploids [4, 5]. Each amphidiploid was expected to contain the complete genome of wheat plus the complete genome of the wild relative. However, chromosome analysis of a number of the amphidiploids using genomic *in situ* hybridization (GISH) showed some variation in chromosome number of both the wheat genome and that of the wild relative. A complete GISH analysis of all the lines used in the experiments was not possible and therefore the present analysis will consider each amphidiploid as an individual genotype.

**Table 1:** Eighteen amphidiploid lines with the wild parent accessions and bread wheat parents used to produce the amphidiploid lines.

Code	Bread wheat parent (AABBDD)	Wild relative parent accession No.	Genome(s)	Abbreviations
1	Chinese Spring	*Amblyopyrum muticum* 2130004	2n = 2x = 14 (TT)	*Am*. *mut*4 x CS
2	Chinese Spring	*Amblyopyrum muticum* 2130008	2n = 2x = 14 (TT)	*Am*. *mut*8 x CS
3	Paragon	*Amblyopyrum muticum* 2130012	2n = 2x = 14 (TT)	*Am*. *mut*12 x PAR
4	Highbury	*Amblyopyrum muticum* 2130012	2n = 2x = 14 (TT)	*Am*. *mut*12 x HB
5	Chinese Spring	*Amblyopyrum muticum* 2130012	2n = 2x = 14 (TT)	*Am*. *mut*12 x CS
6	Chinese Spring	*Aegilops speltoides* 2140008	2n = 2x = 14 (SS)	*Ae*. *spe*8 x CS
7	Pavon 76	*Aegilops speltoides* 2140008	2n = 2x = 14 (SS)	*Ae*. *spe*8 x PAV
8	Pavon 76	*Aegilops speltoides* 449340	2n = 2x = 14 (SS)	*Ae*. *spe*40 x PAV
9	Pavon 76	*Aegilops umbellulata* 542377	2n = 2x = 14 (UU)	*Ae*. *umb*77 x PAV
10	Chinse Spring	*Aegilops umbellulata* 554410	2n = 2x = 14 (UU)	*Ae*. *umb*10/0 x CS
11	Chinse Spring	*Aegilops umbellulata* 554410	2n = 2x = 14 (UU)	*Ae*. *umb*10/3 x CS
12	Paragon	*Aegilops comosa var*. *comosa* 276970	2n = 2x = 14 (MM)	*Ae*. *com*70 x PAR
13	Chinese Spring	*Thinopyrum turcicum* P208/201	2n = 10x = 70 (EE)	*Th*. *tur*201 x CS
14	Highbury	*Secale anatolicum* P208/142	2n = 2x = 14 (RR)	*Se*. *ana*142 x HB
15	Chinese Spring	*Secale anatolicum* P208/142	2n = 2x = 14 (RR)	*Se*. *ana*142 x CS
16	Chinese Spring	*Secale anatolicum* P208/141	2n = 2x = 14 (RR)	*Se*. *ana*141 x CS
17	Chinese Spring	*Secale anatolicum* P208/142	2n = 2x = 14 (RR)	*Se*. *ana*142/ x CS
18	Chinese Spring	*Thinopyrum bessarabicum*	2n = 2x = 14 (JJ)	*Th*. *bes* x CS
CSE	Chinese Spring		2n = 6x = 42 (AABBDD)	CS
PAR	Paragon		2n = 6x = 42 (AABBDD)	PAR
HB	Highbury		2n = 6x = 42 (AABBDD)	HB
PAV	Pavon 76		2n = 6x = 42 (AABBDD)	PAV

### 2.2. Experimental treatments and design

Two glasshouse experiments were conducted at the University of Nottingham, UK, Sutton Bonington Campus. The experiments were sown on 23 June 2014 and 6 July 2015 and harvested on 15 December 2015 and 28 December 2016. The experimental procedures and measurements were the same in both years unless stated otherwise. Seeds were sown in plastic modules filled with soil compost (Levington Advance Seed and Modular F2S). After seed germination (~6 days after sowing) seedlings of 2–4 cm length were transferred to a cold room for vernalization for eight weeks at 6°C with a 12 h photoperiod and then transplanted into 2 L pots (16.8 cm diameter) in the glasshouse using low N peat-compost (Klasmann Medium Peat 818) supplemented with nutrients as described in S1 Table.

The experimental design used was a split-plot where two levels of N treatment (HN: high N and LN: low N) were randomized on the main-treatment and 22 genotypes (18 amphidiploids and four bread wheat parents) were randomized on the sub-plot treatment. There were four replicates. A single seedling was transplanted per pot and represented one replicate. Nitrogen was applied as ammonium nitrate (NH_4_NO_3_) granules (34% N) dissolved in water. Two levels of N were applied, low N at 60 kg ha^-1^ equivalent and high N at 200 kg ha^-1^ equivalent (0.25 and 1.27 g NH_4_NO_3_ pot^-1^ under LN and HN conditions, respectively). For the low N treatment, N application was split into two doses each of 30 kg N ha^-1^ equivalent and for the high N treatment into three doses of 50 kg N ha^-1^, 50 kg N ha^-1^ and 100 kg N ha^-1^ equivalents. The first application was applied immediately after transplanting and the second at onset of stem extension (GS31) for both treatments. The last application for the high N treatment was at flag-leaf emergence (GS39, Zadoks growth stage [29]). The eighteen amphidiploid lines and four bread wheat parents used in the experiment are shown in Table 1.

### 2.3 Glasshouse conditions

Plants were irrigated with a complete nutrient solution (minus N) regularly with an automatic irrigation system to maintain plants free from water stress and nutrient stresses (other than N). The composition of the complete nutrient solution (minus N) is described in S1 Table. Daily minimum and maximum air temperature was measured using a tiny tag temperature data logger (Gemini data loggers, S2 Fig). Supplementary lighting to extend the photoperiod with a light cycle of 16 h at 20°C and dark cycle of 8 h at 15°C was provided in each year from 21 October onwards. Overall accumulated ambient solar radiation during the experiments was 11,501 KJ m^-2^ between June and December in 2014 and 11,743 KJ m^-2^ between July to December 2015 measured on the glasshouse roof.

### 2.4 Plant development and plant height

Regular monitoring of plant growth stages was done following the decimal code of Zadoks growth stages. The growth stages for a plant were assigned when the main shoot was at the specific stage. In both years, heading date (GS55), anthesis date (AD, GS61) and physiological maturity date (PMD, GS89, when the peduncle of the main shoot was 100% senesced) were assessed. Plant height to the tip of the ear (excluding awns) was measured on the main shoot at harvest.

### 2.5 Leaf gas-exchange traits

Gas-exchange measurements were taken on the flag-leaf of each plant on the main shoot using a Li-Cor 6400 XT Portable Photosynthesis System with chlorophyll fluorescence attachment (Li-Cor Biosciences, NE, USA) under HN and LN conditions. Light-saturated photosynthetic rate (A_*max*_) and stomatal conductance (g_s_) of the flag leaf were measured. Measurements were taken on the flag leaf twice a week between 10.00 and 15.00 h from flag-leaf emergence (GS37) to mid-grain filling (GS85). The Li-Cor 6400 settings were: flow rate 400 μmol s^–1^, block temperature 20°C with ambient relative humidity. The sample (cuvette) CO_2_ concentration was set to 400 μmol mol^–1^ and PAR was set to 1500 μmol m^–2^ s^–1^ (10% blue). All parameters were analyzed by taking average values per plant during each of the pre-anthesis and post-anthesis periods.

At anthesis (GS65), the flag leaf area of the main shoot was estimated by measuring the length and width (at the widest) of the flag leaf with a ruler, and then multiplying the product of the length and the width by the correction factor of 0.83 [26].

### 2.6 Flag-leaf visual senescence scoring

Senescence kinetics of the flag leaf were assessed visually for main shoots by recording the leaf percentage green area using a standard diagnostic key based on a scale of 0–10 (10 = 100% senesced), as described by Gaju et al. [26]. Assessments were carried out weekly after anthesis until full flag-leaf senescence. The data were then fitted against thermal time from anthesis (GS65; base temperature of 0°C) using a modified version of an equation with five parameters consisting of a monomolecular and a logistic function [30]. The onset of post-anthesis senescence (VS.Onset;°Cd) was defined as the onset of the rapid phase of senescence and the end of post-anthesis senescence (VS.End;°Cd) as the thermal time when the visual senescence score is 9.5. The senescence parameters were estimated in each plant and then subjected to ANOVA.

### 2.7 Grain yield and NUE component analysis

Plants were harvested at physiological maturity by cutting the whole plant at ground level in each pot. The plant was divided into: i) the main shoot, ii) remaining fertile shoots (those with an ear) and iii) infertile shoots (those without an ear). Shoots for each of the main-shoot and remaining-fertile-shoot category were divided into: i) ear, ii) leaf lamina and iii) stem and leaf sheath and each component weighed after oven drying at 80°C for 48 h. After drying, ears were threshed and the grain weighed and counted. Plant N% of: i) grain ii) leaf lamina and iii) stem and leaf sheath for each of the main-shoot and remaining-fertile-shoot categories was determined separately using the Dumas method [31]. The weight of the infertile shoots was recorded after oven drying at 80°C for 48 h. NUE and their components were calculated as per Eqs 1–3 on a per plant basis [32].

$$N‐use\;efficiency\;(NUE)=Grain\;Yield\;DM\;(g\;plant^{-1})/available\;N\;(g\;plant^{-1})$$ Eq 1

$$N‐uptake\;efficiency\;(NUpE)=AGNH\;(g\;plant^{-1})/available\;N\;(g\;plant^{-1})$$ Eq 2

$$N‐utilization\;efficiency\;(NUtE)=Grain\;Yield\;DM\;(g\;plant^{-1})/AGNH\;(g\;plant^{-1})$$ Eq 3

where available N includes N from the N fertilizer solution and the peat-compost (S1 Table).

### 2.8 Statistical analysis

ANOVA for a split-plot experimental design was carried out using GenStat 19th edition. A cross-year ANOVA was applied to analyze N treatment and genotype effects across years and the interaction with year, assuming N treatment and genotype were fixed effects and replicates and year were random effects. Skewed data were transformed and probability values from ANOVA for transformed data were used for significance levels of treatments. Correlation and regression analysis using the mean values across years was carried out using GenStat 19^th^ edition. Biplots were created using R program (https://www.r-project.org/) package FactoMineR.

## 3. Results

There was no significant year × N treatment × genotype interaction for the majority of traits including grain yield, biomass plant^-1^ and N uptake plant^-1^ reflecting that the experimental glasshouse condtions were similar in both years. Therefore, results are presented for the cross-year means.

### 3.1 Grain yield, yield components, anthesis date and plant height

**Fig 1:**
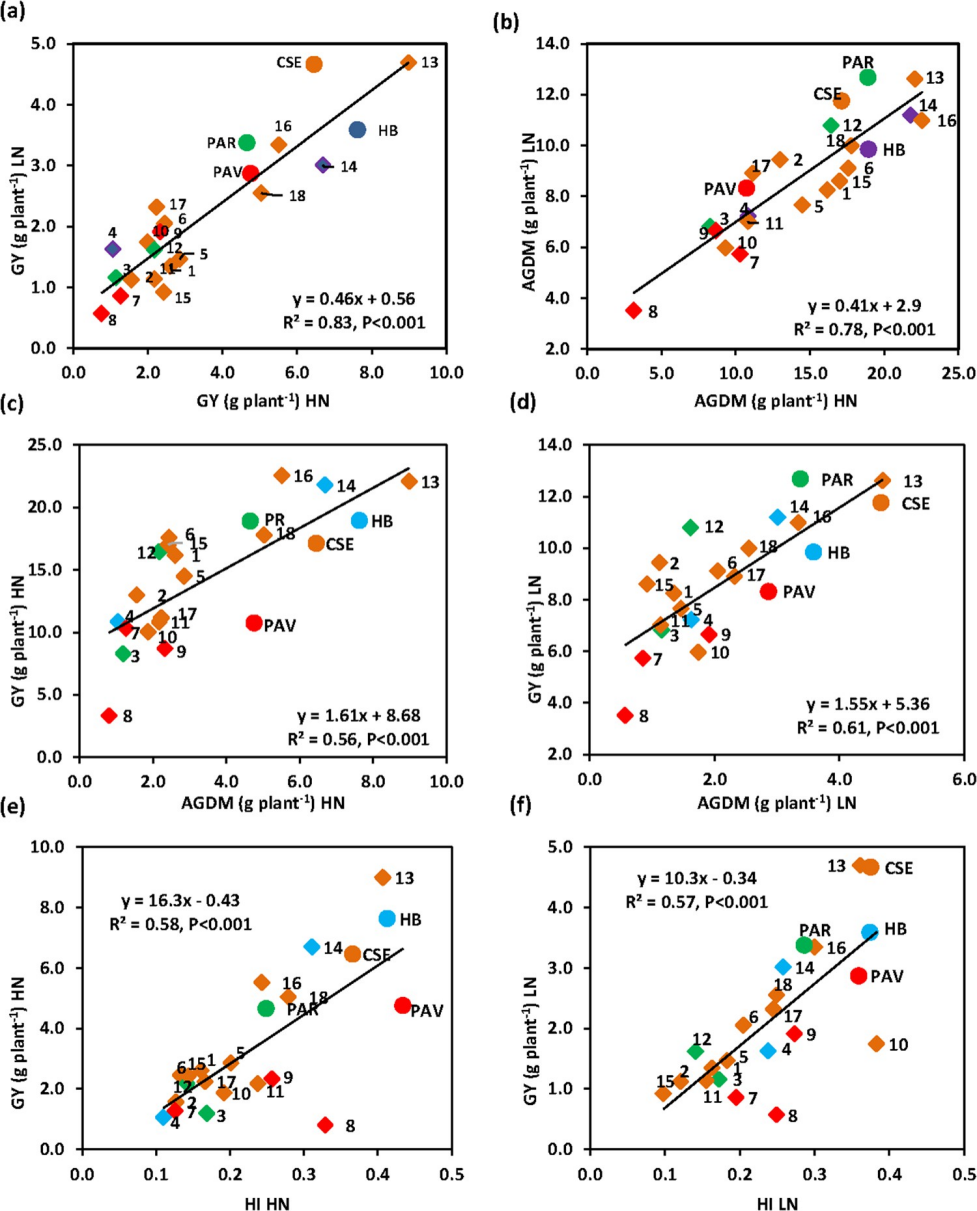
Linear regression of (a) grain yield plant^-1^ (GY, 100% DM) under high N (HN) versus low N (LN), (b) above-ground dry matter plant^-1^ (AGDM, 100%) under HN versus LN, (c) GY versus AGDM under HN, (d) GY versus AGDM under LN, (e) GY versus harvest index (HI) under HN and (f) GY versus HI under LN in 22 genotypes (diagonals: 18 amphidiploid lines and circles: 4 bread wheat parents, same colour used for bread wheat parent and respective amphidiploids) (mean 2015 and 2016). CSE Chinese Spring; HB Highbury; PAR Paragon; PAV Pavon 76. For amphidiploid line codes, see Table 1.

Among the four bread wheat parents, Highbury (HN: 7.63 g plant^-1^) and Chinese Spring (LN: 4.67 g plant^-1^) showed highest grain yield per plant (GY) under HN and LN conditions, respectively. Averaged across years, GY reduced by 38% under LN (2.2 g plant^-1^) compared to HN (3.6 g plant^-1^) conditions (P = 0.004; Table 2; Fig 1A). The genotypes ranged from 0.8 (*Ae*. *spe*40 × Pav) to 9.0 g plant^-1^ (*Th*. *tur*201 × CS) under HN and 0.6 (*Th*. *tur*201 × CS) to 4.7 g plant^-1^ (*Ae*. *spe*40 × Pav) under LN conditions (P<0.001). The reduction under LN ranged from 35% (*Am*. *mut*12 x HB) to 62% (*Se*. *ana*142 × CS) (P< 0.001, Table 2). Overall, there was a positive linear relationship among the genotypes for GY under HN versus GY under LN conditions (R^2^ = 0.83; P<0.001; Fig 1A). The amphidiploid line *Th*. *tur*201 × CS showed significant transgressive segregation (TS) above CS under HN for GY plant^-1^ but there was no TS for any line under LN conditions. Nine lines showed TS for the response of GY to N with relatively less yield reduction under low N than their bread wheat parent. GY was positively associated with HI among genotypes under both N treatments (Fig 1E and 1F).

**Table 2:** Grain yield (GY) plant^-1^, above-ground dry matter (AGDM) plant^-1^, plant height (PH), anthesis date (AD), physiological maturity date (PMD) and ears plant^-1^ in 22 genotypes (18 amphidiploid lines and 4 bread wheat parents) under HN and LN conditions (mean 2015 and 2016).

	GY (g plant^-1^)		AGDM (g plant^-1^)		PH (cm)		AD (DAS)		PMD (DAS)		Ears (plant^-1^)	
	HN	LN	HN	LN	HN	LN	HN	LN	HN	LN	HN	LN
**Chinese Spring**	6.45	4.67	17.1	11.8	82.2	86.8	88	85	132	119	9.88	4.88
Am. mut4 x CS	2.60	1.35	16.2	8.3	80.4	84.2	94	95	140	134	10.00	4.38
Am. mut8 x CS	1.56	1.12	13.0	9.4	73.1	83.6	90	88	139	126	8.00	4.38
Am. mut12 x CS	2.85	1.46	14.5	7.7	79.7	83.5	89	88	141	131	8.88	4.25
Ae. spe8 x CS	2.44	2.05	17.6	9.1	80.9	83.5	92	90	140	127	14.40	6.00
*Ae*. *umb*10/0 x CS	1.87	1.74	10.0	6.0	63.8	55.3	110	106	150	135	9.30	5.29
*Ae*. *umb*10/3 x CS	2.17	1.14	10.8	7.0	67.7	72.8	97	96	140	130	12.3	6.38
*Th*. *tur*201 x CS	8.99 T	4.69	22.1 T	12.6	84.2	88.0	86	84	128	119	12.5	5.50
*Se*. *ana*142 x CS	2.42	0.92	17.0	8.6	85.0	77.2	99	101	139	139	8.38	4.13
*Se*. *ana*141 x CS	5.52	3.34	22.5 T	11.0	105.8 T	108.6 T	89	88	136	122	8.88	3.13
*Se*. *ana*142/ x CS	2.23	2.32	11.2	8.9	74.9	88.3	93	91	149	134	7.75	3.50
Th. bes. x CS	5.03	2.55	17.8	10.0	93.3 T	89.5	91	91	150	139	6.75	3.75
**Paragon**	4.65	3.38	18.9	12.7	67.0	74.3	89	88	134	128	6.50	3.88
Am. mut12 x Par	1.20	1.16	8.3	6.8	57.0	51.6	106	103	141	139	5.48	2.88
Ae. com70 x Par	2.17	1.61	16.4	10.8	58.4	60.4	114	104	162	146	7.63	6.13
**Highbury**	7.63	3.59	18.9	9.9	60.9	62.0	87	86	131	129	6.88	2.88
Am. mut12 x HB	1.06	1.63	10.8	7.2	59.9	60.8	102	104	163	141	5.75	2.63
Se. ana142 x HB	6.69	3.01	21.8 T	11.2	85.2 T	83.9 T	99	99	146	133	6.50	2.75
**Pavon 76**	4.76	2.87	10.7	8.3	57.5	66.4	86	86	133	124	6.50	3.38
Ae. spe8 x Pav	1.27	0.86	10.3	5.7	44.1	52.5	94	96	149	142	11.8	5.13
Ae. spe40 x Pav	0.81	0.57	3.3	3.5	42.3	46.8	95	91	137	135	4.25	3.50
Ae. umb77 x Pav	2.33	1.91	8.7	6.7	59.1	61.9	84	82	129	123	8.25	4.75
Mean	3.56	2.19	14.4	8.8	71.1	74.1	94	93	141	131	8.58	4.30
	SED, Prob		SED, Prob		SED, Prob		SED, Pro		SED, Prob		SED, Pro	
N (6 df)	0.15 ***		0.27 ***		0.80 *		0.31 *		0.81 ***		0.23 ***	
G (260 df)	0.44 ***		1.01 ***		3.85 ***		1.90 ***		2.59 ***		0.71 ***	
N*G (260 df)	0.62 *		1.42 ***		5.40		2.60		3.67 **		1.00 ***	
Y*N*G (260 df)	0.90		2.10		7.55 ***		3.60		5.10 ***		1.44 ns	

Significant at 5%

*, 1%

** and 0.1%

*** level. T indicates transgressive segregation (P< 0.05). DAS, days after sowing.

The above-ground dry matter (AGDM) plant^-1^ was reduced by 39% under N limitation (P<0.001; Table 2). Genotypes ranged from 3.1 (*Ae*. *spe*40 × Pav) to 22.5 (*Se*. *ana*141 × CS) g plant^-1^ under HN and from 3.5 (*Ae*. *spe*40 × Pav) to 12.7 (Paragon) g plant^-1^ under LN conditions (P<0.001). Amphidiploid responses to N limitation ranged from 5.9% (*Ae*. *spe*40 × Pav) to -51.3% (*Se*. *ana*141 × CS) (P< 0.001; Fig 1B). Three lines (*Th*. *tur*201 × CS, *Se*. *ana*142 x HB and *Se*. *ana*141 × CS) showed significant TS above their bread wheat parent under HN. There were no lines with TS under LN conditions (Fig 1B). The three lines (*Th*. *tur*201 × CS, *Se*. *ana*142 × HB and *Se*. *ana*141 × CS) with the highest grain yield under HN conditions each also showed significant TS over their bread wheat parents for AGDM plant^-1^ under HN conditions (Fig 4). Three lines (*Am*. *mut*12 × Par, *Am*. *mut*12 x HB and *Se*. *ana*142/ × CS) showed significantly lower reductions in biomass under N limitation compared to their bread wheat parents.

Anthesis date (AD) was advanced by 1 day and physiological maturity date (PMD) by 10 days under LN compared to HN conditions (Table 2). AD ranged from 84–114 DAS under HN and 82–106 DAS under LN conditions (P<0.001). However, there was no TS for AD amongst genotypes. There was a negative association amongst genotypes for each of AD (HN: P = 0.02; LN: P = 0.01) and PMD (HN: P = 0.02; LN: P < 0.001) with GY plant^-1^ under HN and LN conditions, but no associations with AGDM plant^-1^. Plant height (PH) increased slightly from 71 cm under HN to 74 cm under LN conditions (P = 0.033; Table 2) and ranged amongst the genotypes from 42.3 (*Ae*. *spelt*40 × Pav) to 105.8 cm (*Se*. *ana*141 × CS) under HN and 46.8 (*Ae*. *spelt*40 × Pav) to 108.7 cm (*Se*. *ana*141 × CS) under LN conditions (P<0.001). Three lines under HN and two lines under LN conditions showed TS for increased PH compared to their bread wheat parent (Table 2). Plant height was positively associated with GY plant^-1^ (HN: P = 0.02; LN: P = 0.02) and AGDM plant^-1^ (HN: P <0.001; LN: P <0.001) in both N treatments.

There was a significant reduction in ears plant^-1^ under LN conditions (-50.3%; Table 2; P = 0.002; Table 2). Genotypes ranged from 4.3 (*Ae*. *spe*40 x Pav) to 14.4 (*Ae*. *spe*8 × CS) plant^-1^ under HN and from 2.6 (*Am*. *mut*12 × HB) to 6.4 (*Ae*. *umb*10/3 × CS) ears plant^-1^ under LN conditions (P<0.001). The reduction in ears plant^-1^ under LN ranged from 17.7 to 64.8% (P< 0.001). Five lines under HN and four lines under LN conditions showed positive TS compared to their bread wheat parent.

### 3.2 Leaf photosynthesis traits

#### Flag-leaf photosynthesis rate and stomatal conductance

**Fig 2:**
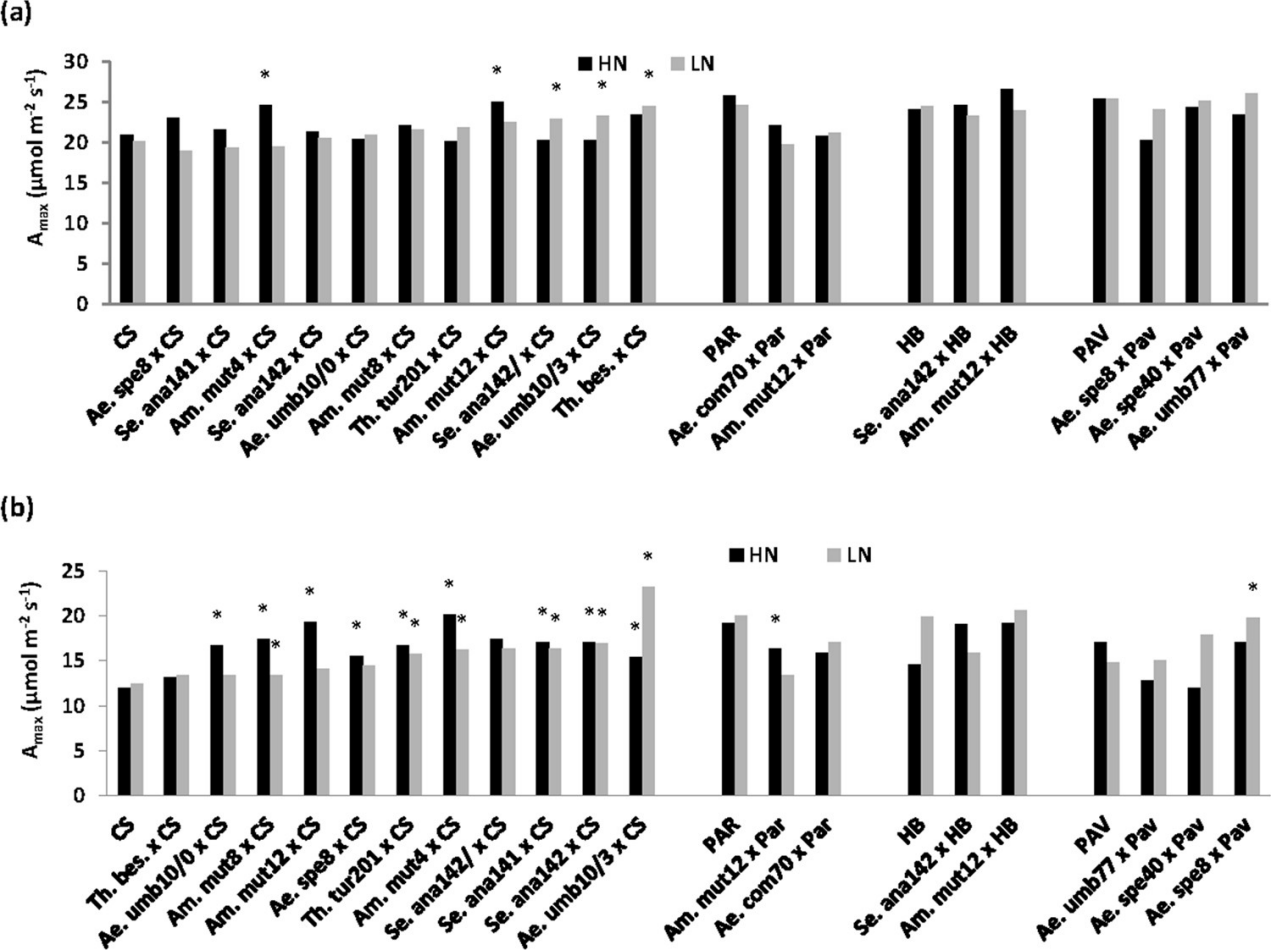
**(a)** Pre-anthesis flag-leaf photosynthesis rate (Pre-An A_max_) and **(b)** post-anthesis flag-leaf photosynthesis rate (Post-An A_max_) for 22 genotypes (18 amphidiploid lines and 4 bread wheat parents) under HN and LN conditions (mean 2015 and 2016) (* indicates transgressive segregation above respective bread wheat parent at 5% level of significance).

There was no significant effect of N on pre-anthesis flag-leaf photosynthesis rate (S2 Table, Fig 2A); genotypes ranged from 20.9 (CS) to 26.6 (*Am*. *mut*12 × HB) μmol m^−2^ s^−1^ under HN and 19.0 (*Ae*. *spe*8 x CS) to 26.1 (*Ae*. *umb*77 × Pav) μmol m^−2^ s^−1^ under LN conditions (P<0.001). Two amphidiploids (*Am*. *mut4* × CS and *Am*. *mut*12 × CS) under HN and three amphidiploids (*Ae*. *umb*10/3 x CS, *Se*. *ana*142 × CS and *Th*. *bes* × CS) under LN showed positive TS for pre-anthesis A_max_ (Fig 2A). There was no association between pre-anthesis A_max_ and flag-leaf area under both HN and LN conditions.

Post-anthesis A_max_ ranged from 12.0 (CS) to 20.2 (*Am*. *mut*4 × CS) μmol m^−2^ s^−1^ under HN and 12.3 (CS) to 23.2 (*Ae*. *umb*10/3 × CS) μmol m^−2^ s^−1^ under LN conditions (P<0.001; S2 Table, Fig 2B). Twelve amphidiploids under HN and seven under LN showed significant positive transgressive segregation above their bread wheat parents (Fig 2B). Also three lines (*Ae*. *spe*8 × PAV, *Ae*. *spe*40 × PAV and *Ae*. *umb*10/3 × CS) maintained post-anthesis A_max_ relatively better under N limitation than their bread wheat parents. There was a positive association between post-anthesis A_max_ and flag-leaf area under HN conditions (R^2^ = 0.42, P = 0.046). The three amphidiploids which showed TS for AGDM plant^-1^ under HN conditions (*Th*. *tur*201 × CS, *Se*. *ana*142 × HB and *Se*. *ana*141 × CS) tended to maintain flag-leaf photosynthesis rate better than their bread wheat parents during late grain filling under HN conditions (S1 Fig).

Flag-leaf pre-anthesis stomatal conductance (g_s_) ranged among genotypes from 0.24 to 0.53 mol m^−2^ s^−1^ under HN and 0.27 to 0.61 mol m^−2^ s^−1^ under LN conditions (S2 Table; P<0.001). One line (*Ae*. *umb*77 × PAV) maintained g_s_ better under N limitation than its bread wheat parent. For post-anthesis g_s_ four lines (*Ae*. *spe*8 × PAV, *Ae*. *spe*40 × PAV, *Ae*. *umb*77 × PAV and *Ae*. *umb*10/3 × CS) maintained g_s_ better under N limitation than their bread wheat parent (S2 Table).

### 3.3 Above-ground N uptake at harvest and N-utilization efficiency

**Fig 3:**
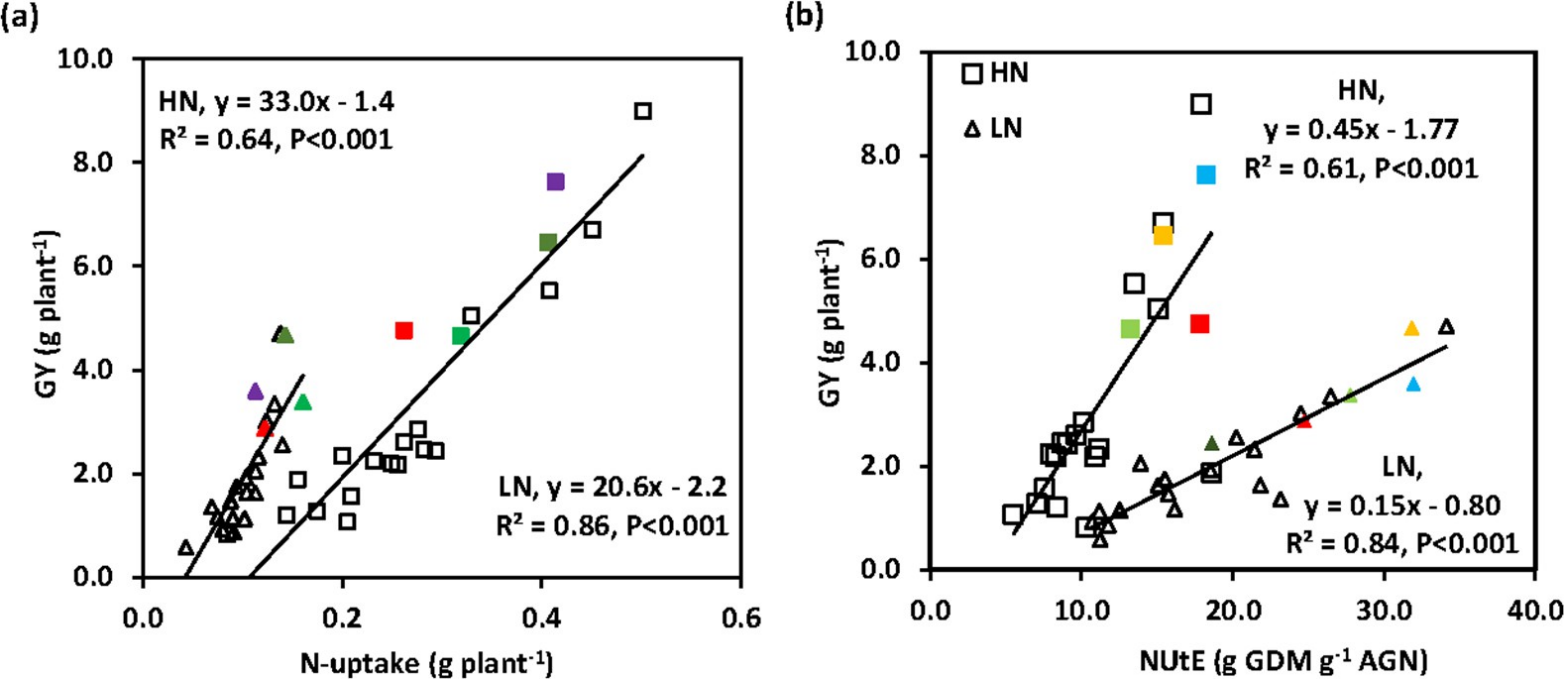
Linear regression of grain yield (GY, 100% DM) on (**a)** N uptake plant^-1^ and **(b)** N-utilization efficiency (g grain dry matter (GDM) per g above-ground N (AGN)) at harvest under high N (◊) and low N (△) conditions in 22 genotypes (18 amphidiploid lines, 4 bread wheat parents, parents in colour symbols (Red: Pavon 76, Blue: Highbury, Green: Paragon, Orange: Chinese Spring) (mean 2015 and 2016).

Above-ground N-uptake at harvest (AGN_H_) was reduced from 0.28 g N under HN to 0.11 g N plant^-1^ under LN conditions (P<0.001; S3 Table). The genotypes ranged from 0.08 (*Ae*. *spe*40 × Pav) to 0.50 (*Th*. *tur*201 × CS) g N plant^-1^ under HN and from 0.04 (*Ae*. *spe*40 × Pav) to 0.16 (Par) g N plant^-1^ under LN conditions (P<0.001). Genetic variation in N uptake plant^-1^ showed a positive association with GY plant^-1^ in both N treatments (Fig 3A). The response of the amphidiploids to LN ranged from -38.7 to -73.7%. There was a positive association amongst genotypes between AGN_H_ and AGDM plant^-1^ in the HN (R^2^ = 0.82, P<0.001) and LN (R^2^ = 0.63, P<0.001) treatment. Two lines (*Th*. *tur*201 × CS and *Se*. *ana*142 × HB) showed positive TS for N uptake plant^-1^ under HN conditions.

Averaing across years, N-utilization efficiency (NUtE) increased from 12.0 under HN to 20.0 g DM g^-1^ N under LN conditions (P<0.001, S3 Table). The genotypes ranged from 5.5 (*Am*. *mut*12 × HB) to 19.5 (*Ae*. *umb*10/0 × CS) g DM g^-1^ N under HN and 10.7 (*Se*. *ana*142 × CS) to 34.1 (*Th*. *tur*201 × CS) g DM g^-1^ N under LN conditions (P<0.001). The increase under LN ranged from -23.0 to 74.8% (P< 0.001). There was no significant TS over the bread wheat parents in either LN or HN conditions or for the response to N limitation. Genetic variation in NUtE showed a positive association with GY plant^-1^ under both HN and LN conditions (Fig 3B). Genetic variation in grain N% was from 2.21% (*Th*. *tur*20 × CS) to 4.74% (*Ae*. *Spe*8 ×Pav) under LN and 3.41% (Paragon) to 4.63% (*Ae*. *spe*40 × Pav) under HN conditions; and for straw N% from 0.46% (*Th*. *tur*20 ×CS) to 1.26% *Ae*. *spe*40 × Pav) and from 1.28% (Highbury) to 2.23% (*Se*. *Ana*142 × CS), respectively (S3 Table).

### 3.4 Genetic variation in flag-leaf senescence parameters

**Fig 4:**
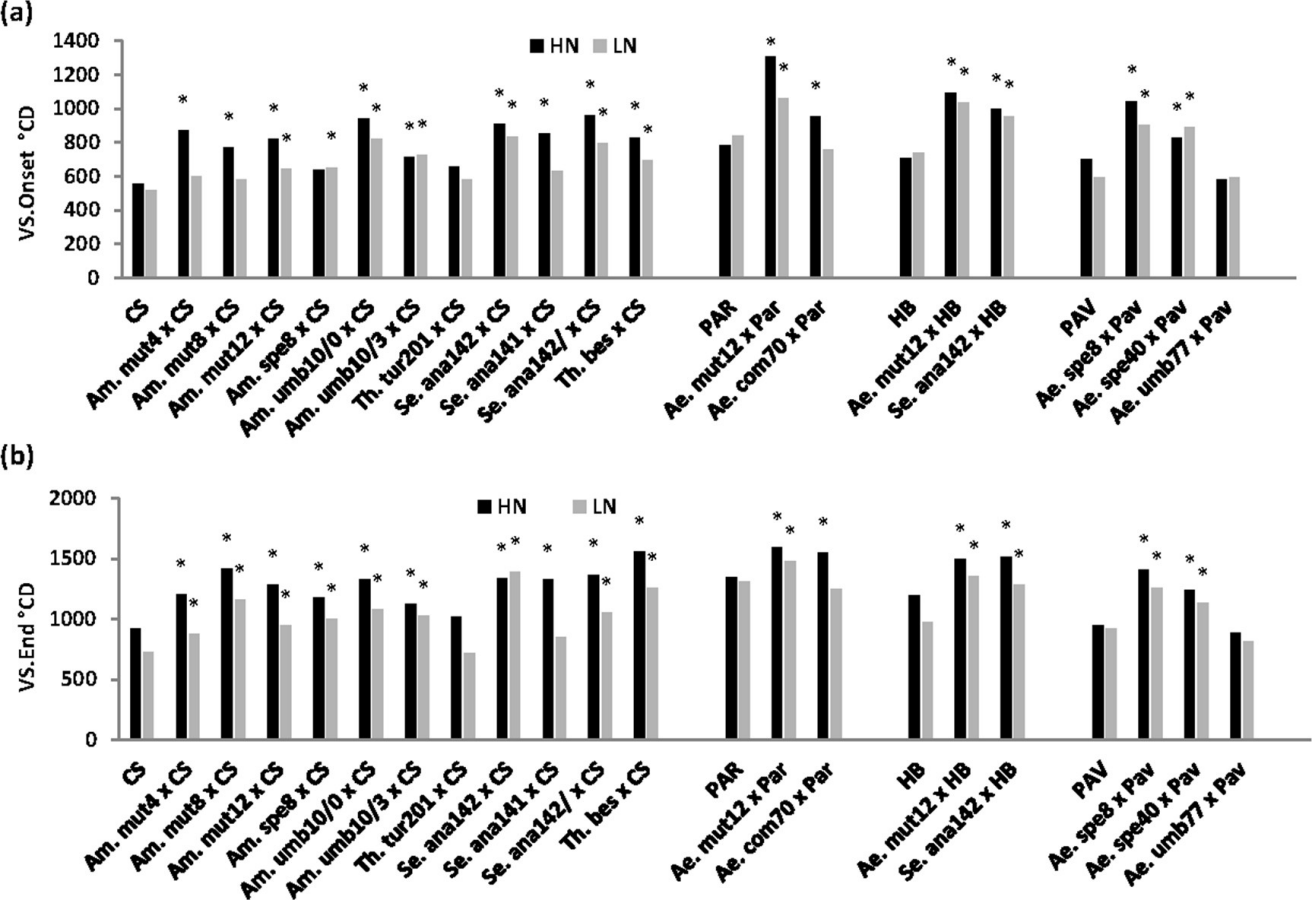
**(a)** Onset (VS.Onset) and **(b)** end (VS.End) of flag leaf sensencence (°Cd post-anthesis (GS61), base temp. 0°C) for 22 genotypes (18 amphidiploid lines and 4 parents) under HN and LN conditions (mean 2015 and 2016). (* indicates transgressive segregation above respective bread wheat parent at 5% level of significance).

Onset of flag-leaf senescence (VS.Onset) was earlier under LN (730.4°Cd post GS65) than under HN (826.6°Cd) conditions (P = 0.016, Table 3). Under HN, VS.Onset ranged from 555.3 (CS) to 1308.7°Cd (*Am*. *mut*12 × Par) and under LN conditions from 519.8 (CS) to 1061.5°Cd (*Am*. *mut*12 × Par) (P<0.001). There was no N × G interaction. Most of the 18 amphidiploid lines showed positive TS with delayed onset of senescence compared to their bread wheat parent under both N treatments (14 under HN and 12 under LN) (Fig 4A). Similar effects were observed for the end of flag-leaf senescence (VS.End) (Table 3; Fig 4B). GY plant^-1^ showed a negative association with VS.Onset under HN conditions and also with VS.End under LN conditions (Table 3).

**Table 3:** Maximum, minimum and mean values for onset (VS.Onset) and end (VS.End) of flag-leaf senescence (thermal time post-anthesis (GS65), base temperature 0°C) for 22 genotypes (18 amphidiploid lines and 4 parents) under HN and LN conditions (mean 2015 and 2016); significance levels for N, genotype (G) and N × G effects; and Pearson’s correlation coefficient for correlation with grain yield (GY) plant^-1^ and above-ground dry matter (AGDM) plant^-1^.

	VS.Onset °Cd		VS.End °Cd	
Genotypes	HN	LN	HN	LN
Min	505.0	378.8	867.4	717.6
Max	1308.7	1061.5	1594.1	1481.7
Mean	826.6	730.4	1265.7	1070.3
Corr. with GY plant^-1^	-0.47 *	-0.38 ns	-0.35 ns	-0.51 *
Corr. with AGDM plant^-1^	-0.22 ns	-0.31 ns	0.05 ns	-0.18 ns
	SED, Df		SED, Df	
N	17.9 * (3.0)		16.3 *** (3.0)	
G	65.9 *** (132)		69.0 *** (116)	
N*G	92.8 ns (134)		96.8 ns (119)	

*Significance at the 5% (P = 0.05) level.

**1% (P = 0.01) level.

***0.1% (P = 0.001) level.

### 3.5 Trait associations for yield, yield components, NUE and flag-leaf traits

**Fig 5:**
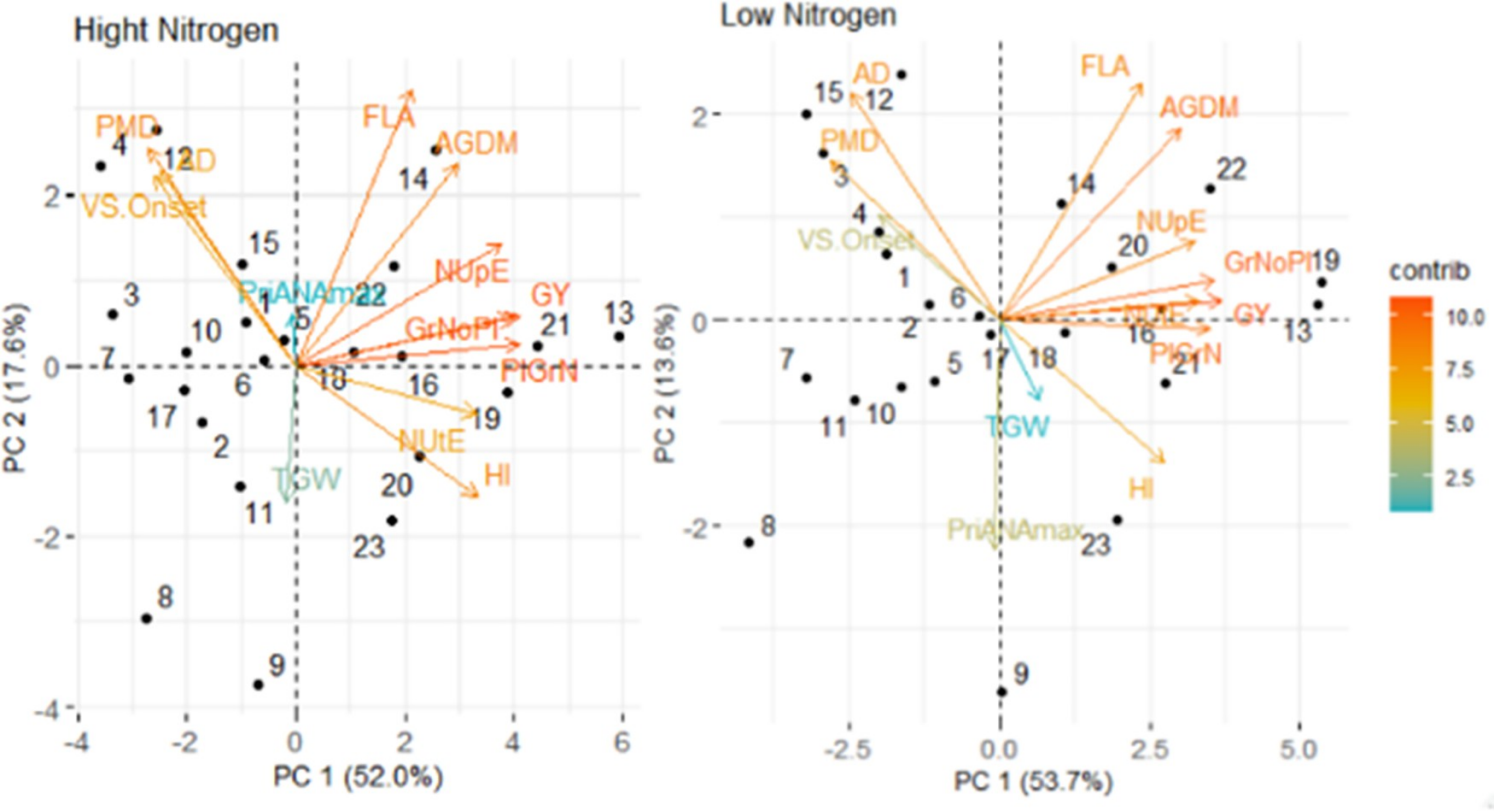
Biplot for 22 genotypes (18 amphidiploid lines and 4 parents) under High N and Low N conditions (mean of 2015 and 2016) for grain yield plant^-1^ (GY), above-ground dry matter plant^-1^ (AGDM), harvest index (HI), days to anthesis (AD), physiological maturity date (PMD), thousand grain weight (TGW), grain number plant^-1^ (GrNoPl), grain N plant^-1^ (PlGrN), N-utilization efficiency (NUtE), N-uptake effciency (NUpE), flag-leaf area (FLA), visual senescence onset (VS.Onset), and pre-anthesis flag-leaf A_max_ (PreANA_max_).

To investigate the trait associations amongst genotypes, principal component analysis was conducted for 13 traits related to grain yield, NUE and flag-leaf senescence for the 18 amphidiploid lines and four bread wheat parents under HN and LN conditions (Fig 5). Under HN, PC1 explained 69.6% of the phenotypic variation and associated traits included GY plant^-1^, grains plant^-1^, grain N plant^-1^ and HI showing a positive effect. PC2 explained 17.6% of variation and associated traits were flag-leaf area and AGDM plant^-1^ with positive effect and TGW with negative effect. Under HN conditions, GY plant^-1^ was positively associated amongst genotypes with grains plant^-1^ (P<0.001), grain N plant^-1^ (P<0.001) and NUpE (P<0.001) and negatively associated with anthesis date and VS.Onset. NUtE showed a positive association with HI and negative association with anthesis date and VS.Onset. In addition, AGDM plant^-1^ showed a positive association with FL area and NUpE and negative association with TGW.

Under LN conditions, PC1 explained 67.3% of the phenotypic variation and the main traits associated were GY plant^-1^, grain N plant^-1^ and grains plant^-1^. PC2 explained 13.6% of variation, the main traits associated with it being pre-anthesis A_max_, while TGW was also associated with a positive effect. GY plant^-1^ showed a positive association with grain N plant^-1^, grains plant^-1^, NUtE and NUpE, and a negative association with AD, PMD and VS.Onset.

### 3.6 Performance of individual amphidiploids showing TS

Among the 18 amphidiploid lines, line *Th*. *tur*201 x CS had the greatest positive transgressive segregation for GY plant^-1^, AGDM plant^-1^ and AGN plant^-1^ under HN conditions. This line also showed the highest TS for NUE and grains plant^-1^ under LN conditions and for post-anthesis flag-leaf A_max_ under both HN and LN conditions (Table 2 and S3 Table). This line, was not significantly taller than its bread wheat parent. Two other amphidiploids, *Se*. *ana*142 × HB and *Se*. *ana*141 × CS, also showed significant TS above their bread wheat parent for AGDM plant^-1^ under HN conditions; however, both these lines were significantly taller than their bread wheat parent under HN conditions.

## 4. Discussion

In the amphidiploid lines, harvest index was lower than their elite bread wheat parents as expected so that grain yield plant^-1^ was generally less than the respective bread wheat parent cultivar. For the same reason, the N-utilization effciency (plant grain dry matter yield / plant N uptake) was generally less in the amphidiploid lines than their respective bread wheat parent cultivars. Therefore, this discussion focuses mainly on genetic variation in the amphidiploids relative to the bread wheats in N uptake plant^-1^ and AGDM plant^-1^ and their physiological basis including leaf photosynthesis rate, assuming that HI can be subsequently increased by modern breeding in material with higher biomass and N uptake.

### 4.1 Effects of plant height and development rate

Plant height under LN (74.1 cm) was slightly higher than under HN (71.1 cm) conditions. Under LN, there was a ca. 50% reduction in fertile shoots plant^-1^ likely resulting in less shading of the main-shoot by tillers than under HN conditions which may have contributed to the small increase in main-shoot plant height under LN. Biomass plant^-1^ increased with increasing PH among genotypes in both N treatments, as has been reported elsewhere [33, 34]. For example, there were genetic gains in plant height in CIMMYT spring wheat cultivars from 1966 to 2009 with a positive association with biomass and grain yield [35]. The amphidiploid lines *Se*. *ana*142 × HB and *Se*. *ana*141 × CS showed TS over their bread wheat parent for PH under LN conditions. Plant height was reported to be positively associated with seedling root length in recent studies on a Savannah × Rialto DH winter wheat population [36] and a winter wheat Avalon × Cadenza DH population [37]. It could be speculated that the taller amphidiploid lines may have had more extensive rooting systems than their parents contributing to increased N uptake and biomass under LN conditions in the present study, although root traits were not presently measured.

Present results showed three amphidiploid lines (*Th*. *tur*201 × CS, *Se*. *ana*142 × HB and *Se*. *ana*141 × CS) had positive TS for biomass plant^-1^ under both HN and LN conditions (except for *Se*. *ana*141 × CS under LN). The biomass increase may be less useful if it is associated with increased PH, as PH is generally fixed in modern wheat breeding programs in the optimum range of ca. 70–100 [38]. Encouragingly, *Th*. *tur*201 × CS had positive TS for biomass plant^-1^ but similar PH to its bread wheat parent and therefore represents promising germplasm for deployment in pre-breeding for biomass improvement.

On average anthesis date was one day earlier and physiological maturity date ten days earlier under LN than HN conditions. Under LN conditions genotypes with earlier anthesis date had higher GY plant^-1^. Earliness is related to the plant’s ability to escape severe abiotic stress conditions [39] by reducing growth pre-anthesis and conserving soil resources for more profitable use in grain growth post-anthesis—a useful strategy under terminal stress conditions. Earlier anthesis amongst genotypes increased GY plant^-1^ due to increased HI rather than biomass consistent with a stress escape effect.

There was a strong association between genetic variation in anthesis date and the timing of flag-leaf senescence in both N treatments, with later anthesis date associated with delayed senescence and extended photosynthesis. Nehe et al. [40] also reported an association between later anthesis and delayed flag-leaf senescence in 16 spring wheat cultivars under HN and LN field conditions in India similar to the present findings, concluding that greater N uptake at anthesis with later anthesis may have buffered N remobilization from flag-leaves contributing to a stay-green effect. Bogard et al. [27], in contrast, reported that delayed leaf senescence (stay-green) was associated with earlier anthesis date and increased grain yield in a winter wheat Toisondor × CF9107 DH population under both high and low N conditions in field experiments in France. However, delayed senescence (stay-green) has showed promise in cereal breeding for improving yield under N deficiency by increase photosynthesis duration [11, 40], linked to optimized post-anthesis N remobilization and N uptake [24, 40]. In the present study the negative association of stay-green with GY under LN conditions may have been also linked to grain sink limitation in the amphidiploid lines [41].

### 4.2 Genetic variation in amphidiploid lines for leaf photosynthesis rate

Pre-anthesis leaf photosynthesis rate was not affected by N treatment. This was likely because there was only a small extent of N stress before anthesis. However, post-anthesis A_*max*_ was also not affected by N treatment implying that flag-leaf N concentration of the main shoot may have been similar in the two N treatments. Significant genetic variation was found in pre- and post-anthesis A_max_ in both N treatments but did not show any association with GY or AGDM plant^-1^. Several amphidiploid lines showed higher leaf photosynthesis rate than their bread wheat parents. Interestingly, the three highest biomass amphidiploid lines (*Th*. *tur*201 x CS, *Se*. *ana*142 x HB and *Se*. *ana*141 x CS) each tended to maintain high post-anthesis A_max_ for longer than their parent cultivars under both N treatments (S1 Fig). There was more TS for post-anthesis A_*max*_ than pre-anthesis A_*max*_ and results showed specific amphidiploid lines had potential to increase leaf photosynthesis rate compared to their bread wheat parent. Encouragingly, there was no negative trade-off between flag-leaf pre-anthesis A_*max*_ and flag-leaf area under both N conditions, inidcating that higher photosynthesis was independent of flag-leaf area. The lack of a GY plant^-1^ association with flag-leaf A_*max*_ implied that grain growth of the genotypes may have been predominantly sink limited as mentioned above. Higher leaf photosynthesis rate was reported in synthetic-derived hexaploid lines compared to the Paragon UK spring wheat parental cultivar in UK field experiments [11]. Greater photosynthetic capacity than bread wheat cultivars and synthetic hexaploid wheat lines was reported in hexaploid triticale, octoploid triticale, and Chinese Spring-rye disomic addition lines with rye chromosomes associated with a Rubisco large subunit gene (at heading and grain-filling stages) and a Rubisco small subunit gene (at grain-filling stage) [42, 43]. Furthermore, Merchuk-Ovnat *et al*. [44] investigated drought-related QTL introgressions from emmer wheat into cultivated wheat and found that yield improvement in introgression lines over their recurrent parent was partly due to enhanced flag-leaf photosynthetic capacity. It can also be speculated that the higher leaf photosynthesis rate and biomass of amphidiploid lines than their bread wheat parents may have been in part related to more chloroplasts per mesophyll cell [45].

### 4.3 Genetic variation in genotypes for N uptake and NUtE

N uptake plant^-1^ at maturity showed a strong association with biomass plant^-1^ under both N treatments indicating the important role of N accumulation in maintaining photosynthetic capacity and biomass. N-utilization efficiency was higher under N limitation as reported elsewhere [11, 46]. Also, as expected there was a negative relation amongst genotypes between genetic variation in NUtE and grain N%.

Amongst the 22 genotypes, the bread wheat parent Paragon showed the highest N uptake plant^-1^ under HN conditions. Under LN conditions *Th*. *tur*201 × CS had the highest N uptake plant^-1^ showing TS over its parent CS for biomass plant^-1^ and NUpE indicating its potential as a source of useful traits for breeding for NUE. As far as we are aware this is the first demonstration of an amphidioloid derived from *Thinopyrum turcicum* showing improved abiotic stress tolerance compared to bread wheat. This line also showed highest GY plant^-1^ and second highest AGDM plant^-1^ and highest NUtE under LN conditions. The high NUtE for this amphidiploid was partly explained by a high NHI.

In the present experiments under HN and LN conditions genetic variation in NUtE showed a negative association with the onset of flag-leaf senescence. This may be partly explained by sink-limitation of grain growth in amphidiploids so that GY did not increase even with enhanced post-anthesis photosynthetic capacity in several of the amphidiploid lines. The trend for a positive association of GY plant^-1^ with timing of onset of flag-leaf senescence observed when considering just the four parent bread wheat cultivars with higher HI under LN conditions also indirectly suggested there was sink limitation in the amphidiploid lines. Alternatively, the stay-green trait in the ampidiploid genotypes may have represented a non-functional stay green phenotype in the present study [47].

There was a N × G interaction for NUtE with some genotypes increasing NUtE relatively more than others under low N, e.g., *Th*. *tur*201 x CS, which also maintained NUE and GY plant^-1^ relatively better under LN conditions. Numerous previous studies of cultivars and segregating populations have shown an inverse relationship between NUtE and grain N% [48, 49], which was also observed amongst the genotypes in our study. Therefore, an enhanced ability to produce viable grains at a low grain N% may be a trait associated with high NUtE and GY under LN conditions. Raising NUtE associated with lower grain N% is feasible in end-use markets for which a high grain starch to protein ratio is desirable, e.g., the feed, distilling or biofuel markets. A lower grain N% and higher NutE may also be linked to a reduced efficiency of post-anthesis N remobilization to the grain [26].

Genetic variation in GY plant^-1^ showed a slightly stronger positive association with N uptake than with NUtE under both HN and LN conditions. Furthermore, the association between GY and N uptake was stronger under LN than under HN conditions. Previous studies in bread wheat also showed N uptake accounted for a greater proportion of genetic variation in NUE under LN than under HN conditions [11, 50–52]. These results indicate that root traits determining N uptake may have been in part underlying the genetic variation in NUE observed under N limitation [53, 54], although root traits were not measured in the present experiments.

Present results showed introducing diversity from wheat wild relatives into cultivated wheat could help in raising NUE in wheat breeding for achieving food security. This supports previous investigations indicating that enlisting wild grass genes is a fesible strategy to combat N limitation in wheat farming, e.g. Subbarao et al. [55]. Several amphidiploids were better adapted to maitianing biomass productivity under low N conditions than their bread wheat parents and therefore have potential for introgressing traits for N stress tolerance in wheat pre-breeding programmes. In the present study we identified amphidiploid lines, e.g. *Th*. *tur*201 x CS, *Se*. *ana*142 x HB and *Se*. *ana*141 x CS, that have potential to be deployed in pre-breeding programmes for higher biomass and NUE under both HN and LN conditions. In future work, these lines need to be backcrossed with elite bread wheat cultivars and the hexaploid derivatives explored further for expression of leaf photosynthesis and N stress tolerance traits to confirm the present results at the field scale.

## Supporting information

S1 Fig Flag-leaf photosynthesis rate (A_max_) against thermal time after onset booting (GS41) for 3 highest yielding amphidiploids lines (a) *Th*. *tur*201 x CS (b) *Se*. *ana*142 x HB and (c) *Se*. *ana*141 x CS and their bread wheat parents under HN and LN conditions in 2015 (LHS, left hand side) and 2016 (RHS).
(DOCX)

S2 Fig Mean daily glasshouse temperature (°C) in 2014–15 and 2015–16. (DOCX)

S1 Table Composition of low N peat-compost and nutrient solution (minus N) used in glasshouse experiments in 2015 and 2016. (DOCX)

S2 Table Pre- and post- anthesis flag-leaf photosynthesis rate (pre-An A_max_ and post-An A_max_), stomatal conductance (pre-An cond. and post-An cond.) and maximum efficiency of PSII (pre-An Fv’/Fm’ and post-An Fv’/Fm’) for 22 genotypes (18 amphidiploid lines and 4 bread wheat parents) under high N (HN) and low N (LN) conditions (mean of 2015 and 2016). (DOCX)

S3 Table N-utilization efficiency for plant (PL NUtE), above-ground N per plant (PL AGN) and N harvest index (NHI) in 22 genotypes (18 amphidiploid lines and 4 bread wheat parents) under high N (HN) and low N (LN) conditions (mean of 2015 and 2016). (DOCX)
