# Supplementary material for: Identifying variation for N-use efficiency and associated traits in amphidiploids derived from hybrids of bread wheat and the genera Aegilops, Secale, Thinopyrum and Triticum
Source: PLoS One. 2022 Apr 15;17(4):e0266924. doi: 10.1371/journal.pone.0266924 (PMC9012389; doi:10.1371/journal.pone.0266924)
Supplement: S1 Fig — Flag-leaf photosynthesis rate (Amax) against thermal time after onset booting (GS41) for 3 highest yielding amphidiploids lines (a) Th. tur201 x CS (b) Se. ana142 x HB and (c) Se. ana141 x CS and their bread wheat parents under HN and LN conditions in 2015 (LHS, left hand side) and 2016 (RHS). (DOCX) [file pone.0266924.s001.docx]

**(a) *Th. tur*201 × Chinese Spring in 2015 and 2016**


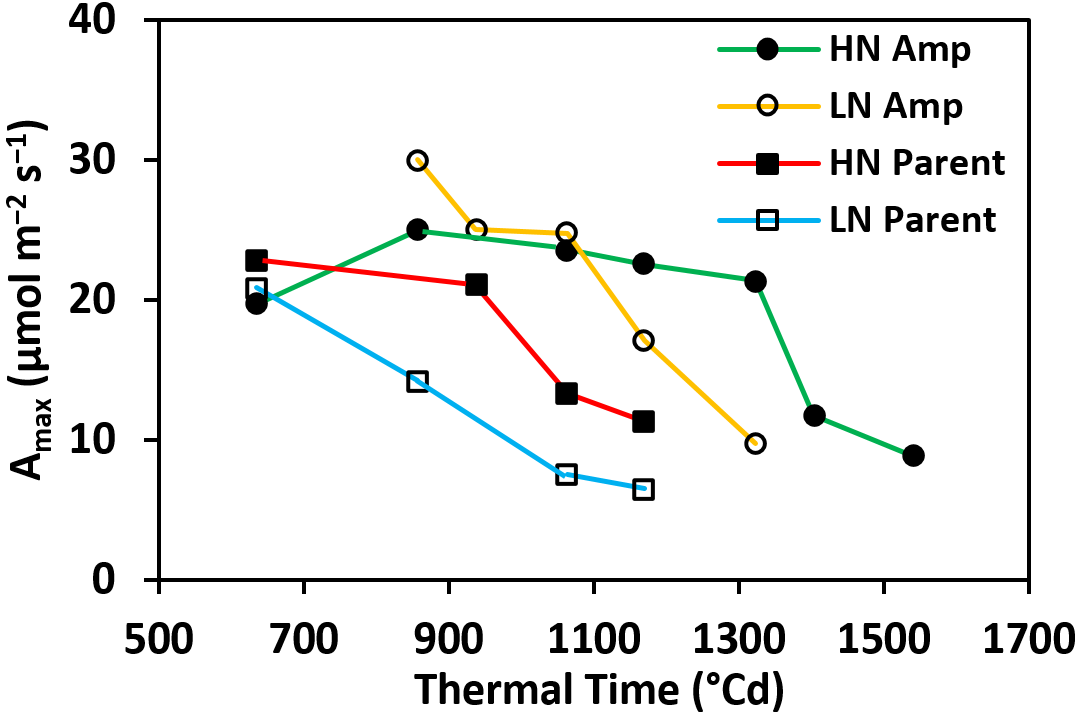

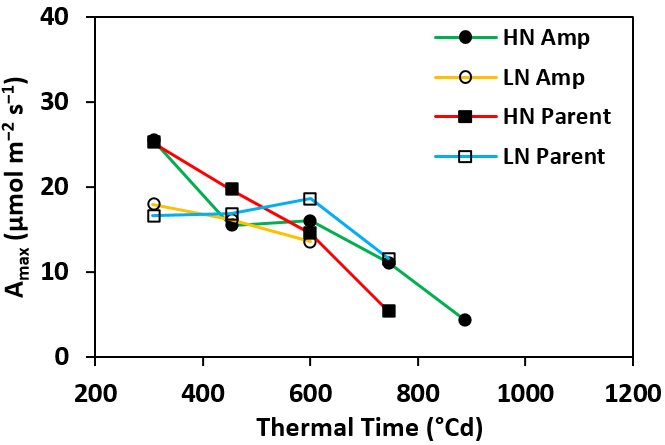


**(b) *Se. ana*142 × Highbury in 2015 and 2016**


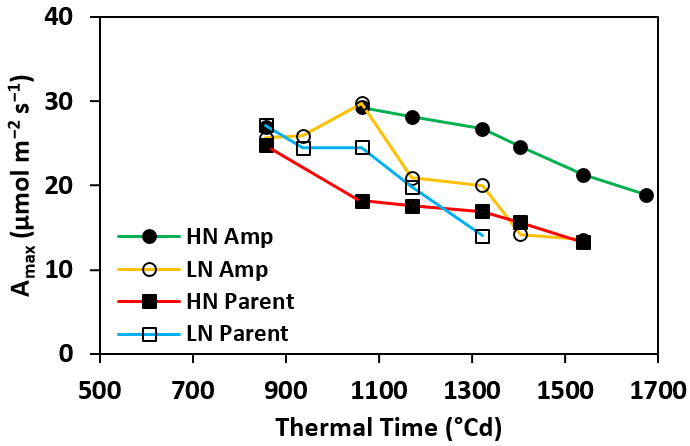

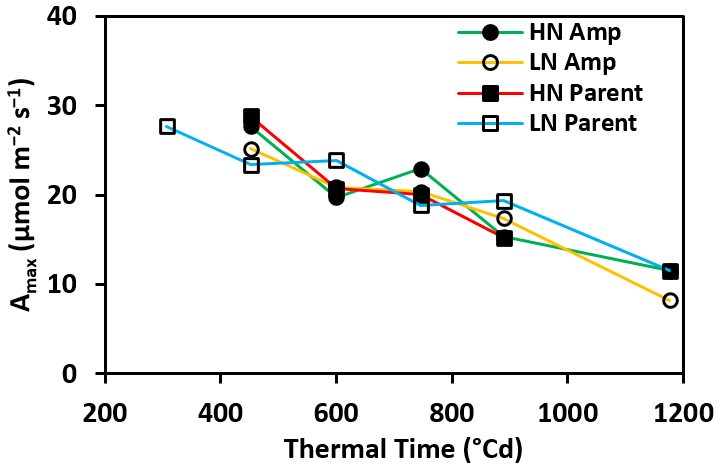


**(c) *Se. ana*141 × Chinese Spring in 2015 and 2016**


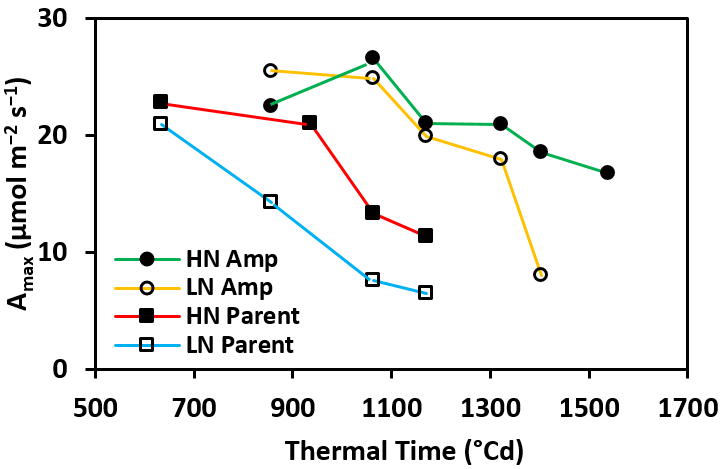

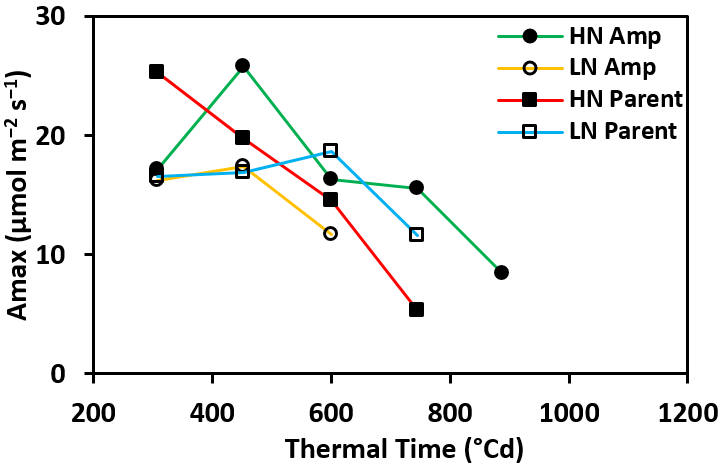


**Figure S1.** Flag-leaf photosynthesis rate (A_max_) against thermal time after onset booting (GS41) for 3 highest yielding amphidiploids lines (a) *Th. tur*201 x CS (b) *Se. ana*142 x HB and (c) *Se. ana*141 x CS and their bread wheat parents under HN and LN conditions in 2015 (LHS, left hand side) and 2016 (RHS)
