# Supplementary figures and images for: Identifying variation for N-use efficiency and associated traits in amphidiploids derived from hybrids of bread wheat and the genera Aegilops, Secale, Thinopyrum and Triticum
Source: PLoS One. 2022 Apr 15;17(4):e0266924. doi: 10.1371/journal.pone.0266924 (PMC9012389; doi:10.1371/journal.pone.0266924)

**
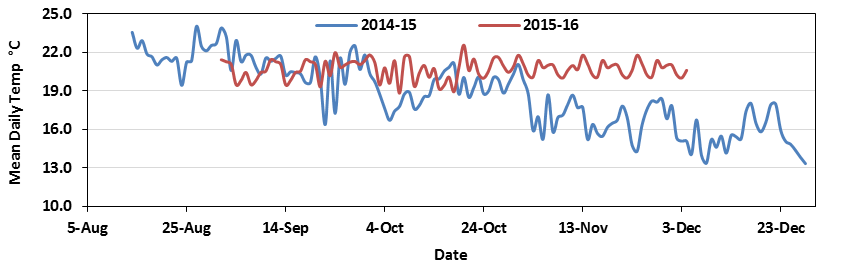
**

**Figure S2**. Mean daily glasshouse temperature (°C) in 2014-15 and 2015-16

Supplement: S2 Fig — (DOCX) [file pone.0266924.s002.docx]
