# Supplementary material for: Identifying variation for N-use efficiency and associated traits in amphidiploids derived from hybrids of bread wheat and the genera Aegilops, Secale, Thinopyrum and Triticum
Source: PLoS One. 2022 Apr 15;17(4):e0266924. doi: 10.1371/journal.pone.0266924 (PMC9012389; doi:10.1371/journal.pone.0266924)
Supplement: S1 Table — (DOCX) [file pone.0266924.s003.docx]

**Table S1.** Composition of low N peat-compost and nutrient solution (minus N) used in glasshouse experiments in 2015 and 2016

| Peat-compost low N | Amount | Units |
| --- | --- | --- |
| Peat: Sand | 100:0 | % volume |
| Single super phosphate | 1.5 | kg m^-3^ |
| Potassium sulphate | 0.75 | kg m^-3^ |
| Ground lime chalk | 2.25 | kg m^-3^ |
| Ground magnesium limestone | 2.25 | kg m^-3^ |
| Fritted trace elements WN225 | 0.4 | kg m^-3^ |
| P_2_O_5_ and K_2_O | 125 and 300 | g m^-3^ = mg litre^-1^ |
| Nutrient solution NPK:0,36,36 | 1 | g per 2 L diluted for a stock solution; 1 ml stock solution/100 ml water |
