# Supplementary material for: Identifying variation for N-use efficiency and associated traits in amphidiploids derived from hybrids of bread wheat and the genera Aegilops, Secale, Thinopyrum and Triticum
Source: PLoS One. 2022 Apr 15;17(4):e0266924. doi: 10.1371/journal.pone.0266924 (PMC9012389; doi:10.1371/journal.pone.0266924)
Supplement: S2 Table — (DOCX) [file pone.0266924.s004.docx]

**Table S2.** Pre- and post- anthesis flag-leaf photosynthesis rate (pre-An A_max_ and post-An A_max_), stomatal conductance (pre-An cond. and post-An cond.) and maximum efficiency of PSII (pre-An Fv’/Fm’ and post-An Fv’/Fm’) for 22 genotypes (18 amphidiploid lines and 4 bread wheat parents) under high N (HN) and low N (LN) conditions (mean of 2015 and 2016)

|  | **Pre-An Cond.**  (mol m^−2^ s^−1^) | | **Pre-An Fv'/Fm'** | | **Post-An Cond.**  (mol m^−2^ s^−1^) | | **Post-An Fv'/Fm'** | | |
| --- | --- | --- | --- | --- | --- | --- | --- | --- | --- |
| **Genotypes** | **HN** | **LN** | **HN** | **LN** | **HN** | **LN** | **HN** | **LN** |  |
|  |  |  |  |  |  |  |  |  |  |
| **Chinese Spring** | 0.30 | 0.36 | 0.55 | 0.52 | 0.16 | 0.17 | 0.50 | 0.52 |  |
| Am. mut4 x CS | 0.39T | 0.29 | 0.58 T | 0.49 | 0.32 T | 0.29 T | 0.56 T | 0.44 |  |
| Am. mut8 x CS | 0.35 | 0.42 T | 0.54 | 0.54 | 0.28 T | 0.22 | 0.53 | 0.50 |  |
| Am. mut12 x CS | 0.45 T | 0.37 | 0.53 | 0.55 | 0.28 T | 0.22 | 0.55 T | 0.47 |  |
| Ae. spe8 x CS | 0.36 | 0.27 | 0.59 T | 0.54 | 0.23 T | 0.30 T | 0.53 | 0.56 |  |
| *Ae. umb*10/0 x CS | 0.30 | 0.44 | 0.55 | 0.55 | 0.22 | 0.17 | 0.57 T | 0.50 |  |
| *Ae. umb*10/3 x CS | 0.37 | 0.50 T | 0.61 T | 0.56 | 0.23 T | 0.49 T | 0.51 | 0.54 |  |
| *Th. tur*201 x CS | 0.24 | 0.35 | 0.54 | 0.57 | 0.24 T | 0.24 | 0.55 T | 0.56 |  |
| *Se. ana*142 x CS | 0.37 | 0.37 | 0.57 | 0.57 | 0.29 T | 0.28 | 0.53 | 0.61 T |  |
| *Se. ana*141 x CS | 0.29 | 0.36 | 0.59 T | 0.54 | 0.25 T | 0.34 T | 0.56 T | 0.50 |  |
| *Se. ana*142/ x CS | 0.27 | 0.40 | 0.57 | 0.55 | 0.29 T | 0.29 T | 0.58 T | 0.52 |  |
| Th. Bes x CS | 0.38 | 0.34 | 0.51 | 0.52 | 0.20 | 0.24 | 0.54 | 0.56 |  |
|  |  |  |  |  |  |  |  |  |  |
| **Paragon** | 0.44 | 0.39 | 0.60 | 0.59 | 0.29 | 0.40 | 0.59 | 0.57 |  |
| Am. mut12 x PAR | 0.35 | 0.31 | 0.54 | 0.54 | 0.31 | 0.21 | 0.58 | 0.50 |  |
| Ae. com70 x PAR | 0.41 | 0.38 | 0.60 | 0.57 | 0.27 | 0.41 | 0.58 | 0.55 |  |
|  |  |  |  |  |  |  |  |  |  |
| **Highbury** | 0.44 | 0.55 | 0.56 | 0.58 | 0.20 | 0.51 | 0.57 | 0.58 |  |
| Am. mut12 x HB | 0.57T | 0.49 | 0.58 | 0.54 | 0.46 T | 0.48 | 0.60 | 0.56 |  |
| Se. ana142 x HB | 0.37 | 0.42 | 0.55 | 0.55 | 0.29 T | 0.32 | 0.55 | 0.54 |  |
|  |  |  |  |  |  |  |  |  |  |
| **Pavon** | 0.49 | 0.51 | 0.59 | 0.64 | 0.28 | 0.26 | 0.58 | 0.54 |  |
| Ae. spe8 x PAV | 0.46 | 0.53 | 0.59 | 0.56 | 0.31 | 0.42 T | 0.61 | 0.62 T |  |
| Ae. spe40 x PAV | 0.37 | 0.45 | 0.58 | 0.57 | 0.17 | 0.37 T | 0.57 | 0.59 T |  |
| Ae. umb77 x PAV | 0.53 | 0.60 T | 0.61 | 0.53 | 0.18 | 0.31 | 0.54 | 0.55 |  |
|  |  |  |  |  |  |  |  |  |  |
| **Mean** | **0.38** | **0.41** | **0.57** | **0.55** | **0.26** | **0.31** | **0.56** | **0.53** |  |
|  | SED | df | SED | Df | SED | Df | SED | df |  |
| N | 0.021 | 3 | 0.01 | 3 | 0.02 * | 5 | 0.01 * | 5 |  |
| G | 0.05 *** | 121 | 0.02 *** | 115 | 0.03 *** | 118 | 0.02 *** | 119 |  |
| N*G | 0.08 | 101 | 0.03 * | 114 | 0.05 *** | 104 | 0.03 *** | 114 |  |
| Y*N*G | 0.09 | 184 | 0.04 ** | 173 | 0.08 * | 51.7 | 0.05 | 48.2 |  |

Significant at 5% *, 1% ** and 0.1% *** level. T shows transgressive segregation.
