# Supplementary material for: Identifying variation for N-use efficiency and associated traits in amphidiploids derived from hybrids of bread wheat and the genera Aegilops, Secale, Thinopyrum and Triticum
Source: PLoS One. 2022 Apr 15;17(4):e0266924. doi: 10.1371/journal.pone.0266924 (PMC9012389; doi:10.1371/journal.pone.0266924)
Supplement: S3 Table — (DOCX) [file pone.0266924.s005.docx]

**Table S3.** N-utilization efficiency for plant (PL NUtE), above-ground N per plant (PL AGN) and N harvest index (NHI) in 22 genotypes (18 amphidiploid lines and 4 bread wheat parents) under high N (HN) and low N (LN) conditions (mean of 2015 and 2016)

|  | **PL NUtE**  **(g DM g^-1^AGN)** | | **PL AGN**  **(g plant^-1^)** | | **NHI** | | **PL Grain**  **N%** | | **PL Straw**  **N%** | |
| --- | --- | --- | --- | --- | --- | --- | --- | --- | --- | --- |
| **Genotypes** | **HN** | **LN** | **HN** | **LN** | **HN** | **LN** | **HN** | **LN** | **HN** | **LN** |
|  |  |  |  |  |  |  |  |  |  |  |
| **Chinese Spring** | 15.4 | 31.9 | 0.41 | 0.14 | 0.64 | 0.73 | 4.36 | 2.32 | 1.45 | 0.53 |
| *Am. mut*4 x CS | 9.7 | 23.1 | 0.26 | 0.07 | 0.27 | 0.30 | 3.50 | 2.28 | 1.63 | 0.75 |
| *Am. mut*8 x CS | 7.6 | 11.2 | 0.21 | 0.10 | 0.23 | 0.45 | 4.07 | 4.18 | 1.75 | 0.83 |
| *Am. mut*12 x CS | 10.1 | 15.8 | 0.28 | 0.09 | 0.45 | 0.64 | 4.60 | 4.15 | 1.59 | 0.56 |
| *Ae. spe8* x CS | 8.8 | 13.9 | 0.28 | 0.11 | 0.27 | 0.48 | 3.88 | 3.29 | 1.55 | 0.81 |
| *Ae. umb*10/0 x CS | 18.6 | 15.5 | 0.16 | 0.10 | 0.48 | 0.63 | 3.93 | 4.20 | 1.64 | 0.82 |
| *Ae. umb*10/3 x CS | 10.9 | 12.5 | 0.25 | 0.09 | 0.39 | 0.47 | 4.57 | 3.99 | 1.91 | 0.98 |
| *Th. tur*201 x CS | 17.9 | 34.1 | 0.50 T | 0.14 | 0.62 | 0.73 | 3.47 | 2.21 | 1.58 | 0.46 |
| *Se. ana*142 x CS | 9.1 | 10.7 | 0.29 | 0.08 | 0.24 | 0.33 | 3.80 | 3.34 | 1.70 | 0.71 |
| *Se. ana*141 x CS | 13.5 | 26.5 | 0.41 | 0.13 | 0.50 | 0.68 | 3.73 | 2.63 | 1.43 | 0.55 |
| *Se. ana*142 x CS | 7.9 | 21.4 | 0.23 | 0.12 | 0.25 | 0.48 | 4.21 | 2.41 | 2.23 | 0.97 |
| *Th. bes.* x CS | 15.0 | 20.2 | 0.33 | 0.14 | 0.53 | 0.69 | 3.60 | 3.08 | 1.34 | 0.58 |
|  |  |  |  |  |  |  |  |  |  |  |
| **Paragon** | 13.3 | 27.8 | 0.32 | 0.16 | 0.43 | 0.64 | 3.41 | 2.68 | 1.42 | 0.67 |
| *Am. mut*12 x PAR | 8.4 | 16.2 | 0.14 | 0.08 | 0.30 | 0.47 | 4.21 | 3.16 | 1.79 | 0.76 |
| *Ae. com*70 x PAR | 8.3 | 15.0 | 0.26 | 0.11 | 0.20 | 0.47 | 3.90 | 3.34 | 1.79 | 0.64 |
|  |  |  |  |  |  |  |  |  |  |  |
| **Highbury** | 18.3 | 32.0 | 0.41 | 0.11 | 0.63 | 0.73 | 3.45 | 2.50 | 1.28 | 0.49 |
| *Am. mut*12 x HB | 5.5 | 21.8 | 0.21 | 0.10 | 0.17 | 0.37 | 4.38 | 2.61 | 1.97 | 1.14 |
| *Se. ana*142 x HB | 15.4 | 24.5 | 0.45 T | 0.12 | 0.54 | 0.65 | 3.50 | 2.75 | 1.47 | 0.56 |
|  |  |  |  |  |  |  |  |  |  |  |
| **Pavon** | 24.7 | 57.5 | 0.26 | 0.12 | 0.61 | 0.67 | 3.46 | 3.00 | 1.54 | 0.80 |
| *Ae. spe*8 x PAV | 11.7 | 44.1 | 0.18 | 0.09 | 0.29 | 0.48 | 4.16 | 4.74 | 1.76 | 1.10 |
| *Ae. spe*40 x PAV | 11.2 | 42.3 | 0.08 | 0.04 | 0.35 | 0.26 | 4.63 | 4.30 | 1.99 | 1.26 |
| *Ae. umb*77 x PAV | 18.5 | 59.1 | 0.20 | 0.10 | 0.47 | 0.65 | 4.29 | 3.61 | 1.75 | 0.90 |
|  |  |  |  |  |  |  |  |  |  |  |
| **Mean** | **12.0** | **20.0** | **0.28** | **0.11** | **0.41** | **0.55** | **3.96** | **3.22** | **1.66** | **0.77** |
|  | SED | df | SED | df | SED | df | SED | df | SED | df |
| N | 0.94 *** | 6 | 0.01 *** | 6 | 0.007 *** | 6 | 0.15** | 6 | 0.07*** | 6 |
| G | 2.22 *** | 257 | 0.021 *** | 262 | 0.06 *** | 262 | 0.44*** | 254 | 0.14*** | 264 |
| N*G | 3.21 ** | 223 | 0.03 *** | 216 | 0.10 | 245 | 0.63 | 246 | 0.21 | 207 |
| Y*N*G | 4.58 * | 249 | 0.04 ** | 255 | 0.11 | 269 | 0.90 | 264 | 0.29** | 254 |

Significant at 5% *, 1% ** and 0.1% *** level. T: transgressive segregation over parents.
